# Reliable measurement of free Ca^2+^ concentrations in the ER lumen using Mag-Fluo-4

**DOI:** 10.1016/j.ceca.2020.102188

**Published:** 2020-05

**Authors:** Ana M. Rossi, Colin W. Taylor

**Affiliations:** Department of Pharmacology, University of Cambridge, Tennis Court Road, Cambridge CB2 1PD, UK

**Keywords:** AM, acetoxymethyl, [Ca^2+^]_m_, medium free [Ca^2+^], CLM, cytosol-like medium, CLM-H, CLM supplemented with HEDTA, CPA, cyclopiazonic acid, DT40-IP_3_R1, DT40 cells expressing rat type 1 IP_3_R, EC_50_, half-maximally effective concentration, ER, endoplasmic reticulum, G-CEPIA1*er*, green-CEPIA indicator targeted to ER lumen, GECI, genetically-encoded Ca^2+^ indicator, *h*, Hill coefficient, IP_3_, inositol 1,4,5-trisphosphate, IP_3_R, IP_3_ receptor, K_D_, equilibrium dissociation constant, RFU, relative fluorescence unit, QAb, quenching antibody, SERCA, sarcoplasmic/endoplasmic reticulum Ca^2+^-ATPase, Acetoxymethyl ester, Ca^2+^ signal, Ca^2+^ indicator, Endoplasmic reticulum, Inositol 1,4,5-trisphosphate receptor

## Abstract

Synthetic Ca^2+^ indicators are widely used to report changes in free [Ca^2+^], usually in the cytosol but also within organelles. Mag-Fluo-4, loaded into the endoplasmic reticulum (ER) by incubating cells with Mag-Fluo-4 AM, has been used to measure changes in free [Ca^2+^] within the ER, where the free [Ca^2+^] is estimated to be between 100 μM and 1 mM. Many results are consistent with Mag-Fluo-4 reliably reporting changes in free [Ca^2+^] within the ER, but the results are difficult to reconcile with the affinity of Mag-Fluo-4 for Ca^2+^ measured *in vitro* (K_D_^Ca^ ∼22 μM). Using an antibody to quench the fluorescence of indicator that leaked from the ER, we established that the affinity of Mag-Fluo-4 within the ER is much lower (K_D_^Ca^ ∼1 mM) than that measured *in vitro*. We show that partially de-esterified Mag-Fluo-4 has reduced affinity for Ca^2+^, suggesting that incomplete de-esterification of Mag-Fluo-4 AM within the ER provides indicators with affinities for Ca^2+^ that are both appropriate for the ER lumen and capable of reporting a wide range of free [Ca^2+^].

## Highlights

• Mag-Fluo-4 loaded into ER by incubation of cells with Mag-Fluo-4 AM reliably reports ER free [Ca^2+^].
• However, the Ca^2+^ affinity of Mag-Fluo-4 determined *in vitro* appears too high to allow measurements of ER luminal [Ca^2+^].
• We use an antibody (QAb) to quench the fluorescence of leaked indicator.
• Using QAb, we show that indicator within the ER has low affinity and sensitivity across a wide range of [Ca^2+^].
• Incomplete de-esterification of compartmentalized indicator allows it to effectively report ER free [Ca^2+^].

## Graphical abstract

**Figure:**
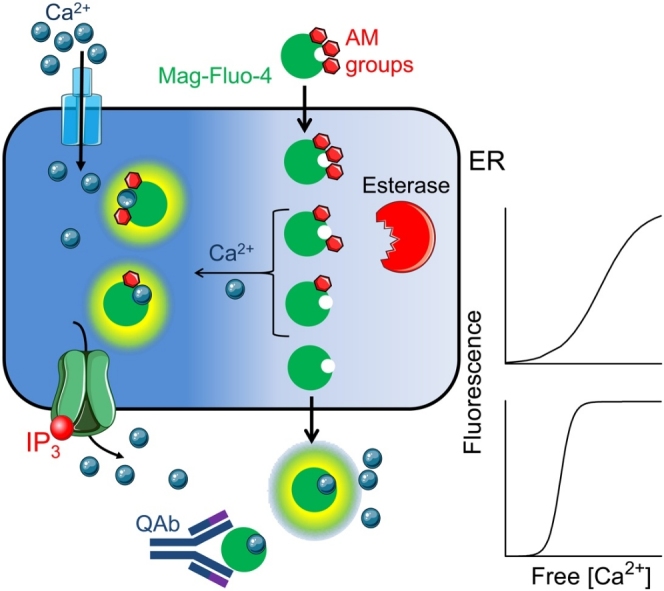

## 1 Introduction

Spatially organized increases in cytosolic free [Ca^2+^] regulate many cellular processes [1,2]. Changes in the free [Ca^2+^] within organelles, notably the endoplasmic reticulum (ER), mitochondria and lysosomes, also regulate cellular activities [3,4]. Many of these signals are initiated by release of Ca^2+^ from the ER through inositol 1,4,5-trisphosphate receptors [5]. There is, therefore, a need for fluorescent Ca^2+^ indicators that can reliably measure free [Ca^2+^] within the cytosol and lumina of intracellular organelles. Both genetically-encoded Ca^2+^ indicators (GECI), derived from endogenous Ca^2+^ sensors [6]; and synthetic Ca^2+^ indicators, derived from carboxylate-based Ca^2+^ buffers [7,8], have been developed to meet these needs. Addition of targeting sequences to GECIs allows selective expression in the ER lumen. Synthetic indicators, which are readily available and usually brighter than GECIs, are often loaded into cells as acetoxymethyl (AM) esters [7]. The AM ester is then cleaved in the cytosol by endogenous esterases that both restore the Ca^2+^-binding site of the indicator and trap the indicator within the cell [7]. Esterified indicators can also cross intracellular membranes; ER-resident carboxylesterases are then assumed to cleave the indicator [9], trapping it within the organelle [10,11]. While this serendipitous compartmentalization of indicator can compromise measurements of cytosolic [Ca^2+^], it can also be exploited to allow measurement of [Ca^2+^] within organelles, like the ER [10].

Several methods, including targeted expression of esterases [12], have been developed to optimize accumulation of synthetic indicators within the ER. Typically, cells are incubated with an esterified indicator under conditions that favour accumulation within the ER, followed by selective permeabilization of the plasma membrane (with digitonin or saponin) to release cytosolic indicator [10,13]. Since the free [Ca^2+^] in the ER lumen is thought to be between 100 μM and 1 mM [[14], [15], [16]], relatively low-affinity indicators are required. Among commercially available Ca^2+^ indicators [17], most have affinities best suited to measuring cytosolic [Ca^2+^] (K_D_^Ca^ < 1 μM). The affinities of Mag-Fura-2 (K_D_^Ca^ = 53 μM) [10] and Fluo-5 N (K_D_^Ca^ ∼90 μM) measured *in vitro*, suggest they would be compatible with measurements of ER [Ca^2+^], but Fluo-5 N can be effectively loaded into the ER only with the aid of ER-targeted esterases [12]. Mag-Fluo-4, a Mg^2+^ indicator, for which the K_D_^Ca^ measured *in vitro* is ∼22 μM [18], has been widely used as an ER Ca^2+^-indicator [13,[19], [20], [21]]. Mag-Fluo-4 is almost non fluorescent in the absence of divalent cations, but its fluorescence increases after binding Ca^2+^ or Mg^2+^. Although Mag-Fluo-4 has been used to measure luminal [Ca^2+^] in the ER with evident reliability [13,[19], [20], [21]], it is puzzling that an indicator with a reported K_D_^Ca^ of 22 μM [18] should not be saturated at the free [Ca^2+^] thought to occur within the ER. An additional concern is that after Mag-Fluo-4 AM is de-esterified within the ER, some indicator leaks into the medium, possibly through organic anion transporters [22]. The problem is negligible with medium free [Ca^2+^] ([Ca^2+^]_m_) designed to mimic an unstimulated cell (*<* 200 nM) because such a small fraction (<1%) of the released indicator binds Ca^2+^, but the leakage becomes more problematic as [Ca^2+^]_m_ approaches or exceeds the K_D_^Ca^.

Here we load the ER with indicator by incubating cells with Mag-Fluo-4 AM and, after permeabilizing the plasma membrane, we use a membrane-impermeant antibody to selectively quench the fluorescence of leaked indicator. This allowed us to reliably determine the affinity of indicator within the ER lumen, and demonstrate that its K_D_^Ca^ is ∼1 mM. We provide evidence that the much reduced affinity of the luminal indicator and its broader range of responsiveness (both useful for monitoring ER [Ca^2+^]) are due to incomplete de-esterification within the ER [23].

## 2 Materials and methods

### 2.1 Materials

Cyclopiazonic acid (CPA) was from Tocris (Bristol, UK). D-myo-inositol 1,4,5- trisphosphate (IP_3_) was from Enzo (Exeter, UK). Synthetic adenophostin A [24] was a generous gift from Prof Barry V. L. Potter, University of Oxford. Mag-Fluo-4 AM, Mag-Fluo-4 tetrapotassium salt, rabbit Fluorescein/Oregon Green polyclonal antibody (QAb, catalogue number A-889), water-soluble probenecid, RPMI medium and Dulbecco's modified Eagle’s medium/nutrient mixture F-12 with GlutaMAX (DMEM/F-12 GlutaMAX) were from ThermoFisher Scientific (Paisley, UK). Plasmid encoding the ER-targeted GECI, G-CEPIA1*er* [25], was from Addgene #58215. DT40 cells lacking endogenous IP_3_Rs were from Dr T. Kurosaki (Kansai Medical University, Japan) [26]. HEK-293 cells were from Kerafast (Boston, USA). TransIT-LT1 transfection reagent was from Geneflow (Elmhurst, Lichfield, UK). G418 was from Formedium (Norfolk, UK). Half-area 96-well black-walled plates were from Greiner Bio One (Stonehouse, UK). Unless otherwise specified, other reagents, including porcine liver carboxylesterase (EC 3.1.1.1) and foetal bovine serum (FBS), were from Sigma-Aldrich (Gillingham, UK).

### 2.2 Cell culture and transfection

Methods used to generate and culture DT40 cells lacking endogenous IP_3_Rs [26] and stably expressing rat IP_3_R1 (DT40-IP_3_R1 cells) were described previously [27]. HEK cells were cultured in DMEM/F-12 GlutaMAX medium with 10 % FBS at 37 °C in 95 % air and 5% CO_2_. Cells were passaged or used for experiments when they reached confluence. To produce HEK cells stably expressing G-CEPIA1*er* (HEK-G-CEPIA1*er* cells), cells were transfected with the G-CEPIA1*er* plasmid [25] using TransIT-LT1 reagent according to the manufacturer’s instructions. To generate stable cell lines, cells were passaged after 48 h in medium with G418 (1 mg/mL) and selection was maintained for 2 weeks, with the medium changed every 3 days. To obtain polyclonal HEK-G-CEPIA1*er* cells, cells were sorted using fluorescence activated cell sorting (FACS) and cells with the highest levels of G-CEPIA1*er* fluorescence (top ∼1%) were isolated and further expanded. All cell lines were confirmed to be free of mycoplasma.

### 2.3 Measurements of Ca^2+^ uptake into and release from the ER

**Fig. 1:**
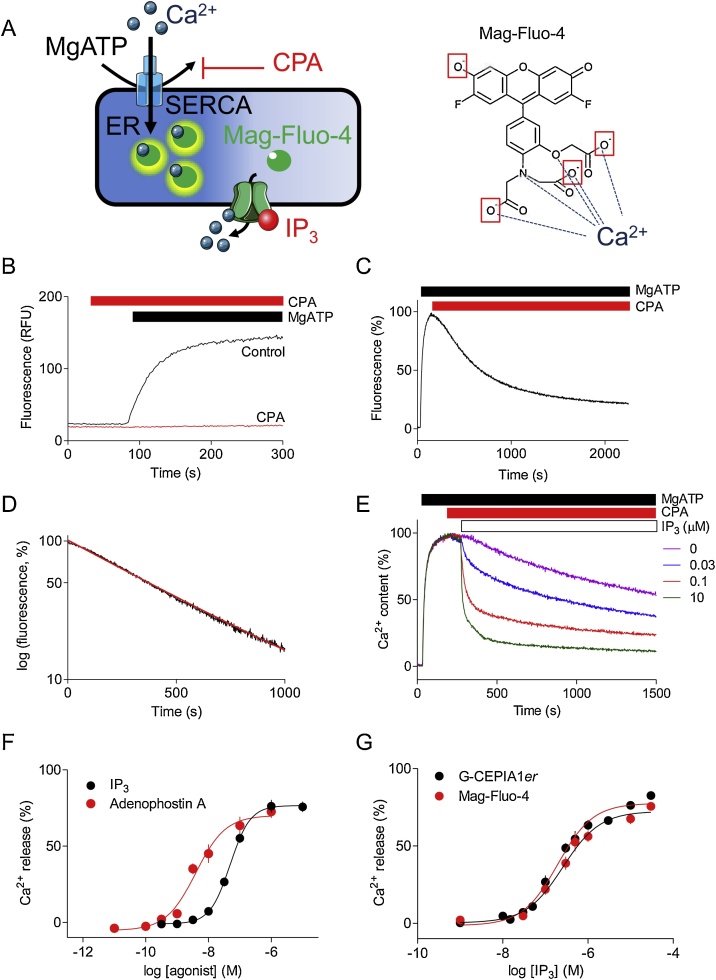
Mag-Fluo-4 within the ER can reliably measure Ca^2+^ uptake and release. (A) Principle of the assay and structure of Mag-Fluo-4 highlighting the groups esterified in Mag-Fluo-4 AM (boxes) and the substituents that coordinate Ca^2+^ (arrows) [40]. (B) The increase in fluorescence from Mag-Fluo-4 after addition of MgATP (1.5 mM) to permeabilized DT40-IP_3_R1 cells is abolished by CPA (10 μM). Traces are typical of 3 independent experiments. RFU, relative fluorescence units. (C) Addition of CPA (10 μM) to permeabilized DT40-IP_3_R1 cells loaded to steady state with Ca^2+^ unmasks a slow Ca^2+^ leak. Trace is the average of 3 independent experiments. (D) Fluorescence after addition of CPA plotted semi-logarithmically (mean ± SEM, *n* = 3). (E) Permeabilized DT40-IP_3_R1 cells loaded with Mag-Fluo-4 AM were stimulated with MgATP (1.5 mM) to fuel Ca^2+^ uptake, CPA (10 μM) to inhibit SERCA, and IP_3_ as indicated. Traces are typical of 3 experiments. (F) Concentration-dependent effects of IP_3_ and adenophostin A (mean ± SEM, *n* = 3, each with 3 replicates). (G) Concentration-dependent effects of IP_3_ on Ca^2+^ release from the ER of permeabilized HEK cells stably expressing G-CEPIA1*er* (HEK- G-CEPIA1*er*) or loaded with Mag-Fluo-4 AM. Means ± SEM from 6-9 independent experiments, each performed in duplicate. Results are summarized in Table 1.

Mag-Fluo-4 was used to monitor free [Ca^2+^] within the ER lumen [28]. The ER was loaded with indicator by incubating cells with Mag-Fluo-4 AM (20 μM, 60 min, 22 °C) in HEPES-buffered saline (HBS: 135 mM NaCl, 5.9 mM KCl, 11.6 mM HEPES, 1.5 mM CaCl_2_, 11.5 mM glucose, 1.2 mM MgCl_2_, pH 7.3), supplemented with BSA (1 mg/mL) and pluronic acid (0.02 % v/v), as previously described [20]. After washing and permeabilization with saponin (10 μg/mL, 37 °C, 2−3 min) in Ca^2+^-free cytosol-like medium (Ca^2+^-free CLM), cells were centrifuged (650 x*g*, 3 min), and re-suspended in Mg^2+^-free CLM supplemented with CaCl_2_ to give a final free [Ca^2+^] of 220 nM after addition of 1.5 mM MgATP. We estimate the free [Mg^2+^] after addition of MgATP to be ∼300 μM, too low to appreciably affect the fluorescence of Mag-Fluo-4 (K_D_^Mg^ =4.7 mM, [18]). We note that all Mag-Fluo-4 fluorescence changes reported herein are due to Ca^2+^ (rather than Mg^2+^) binding because the signals were entirely dependent on addition of MgATP, abolished by inhibition of the ER Ca^2+^ pump (SERCA) by CPA (see Fig. 1B), and SERCA does not transport Mg^2+^ [29]. Ca^2+^-free CLM comprised: 20 mM NaCl, 140 mM KCl, 1 mM EGTA, 20 mM PIPES, 2 mM MgCl_2_, pH 7.0. Cells (∼4 × 10^5^ cells/well) were attached to poly-L-lysine-coated 96-well black-walled plates, and fluorescence (excitation 485 nm, emission 520 nm) was recorded at intervals of 1.44 s using a FlexStation III plate-reader (Molecular Devices, Sunnyvale, CA, USA). MgATP (1.5 mM) was added to initiate Ca^2+^ uptake, and when the ER had loaded to steady state with Ca^2+^ (∼150 s), ligands were added. The same methods were used for HEK-G-CEPIA1*er* cells. Where probenecid (2.5 mM) was used, it was present during dye-loading, permeabilization and fluorescence measurements.

### 2.4 Quenching fluorescence of leaked indicator using an antibody

Permeabilized HEK cells loaded with Mag-Fluo-4 AM were re-suspended (7.5 × 10^6^ cells/mL) in CLM supplemented with 1 mM HEDTA (CLM-H) with appropriate free [Ca^2+^] and either anti-fluorescein/Oregon Green antibody (QAb, 1:20) or control medium (93.5 mM K_2_HPO_4_, 6.5 mM KH_2_PO_4_, 5 mM NaN_3_, pH 8). Free [Ca^2+^] were estimated using MaxChelator [30]. Mag-Fluo-4 fluorescence was measured using a FlexStation III in either the cells (Section 2.3) or in supernatants after centrifugation (650 x*g*, 3 min).

### 2.5 Measurements of Ca^2+^ affinities of indicators within the ER

Permeabilized HEK cells loaded with Mag-Fluo-4 AM or stably expressing G-CEPIA1*er* were incubated with CPA (10 μM, 30 min) to inhibit SERCA and empty the ER Ca^2+^ stores. Cells were re-suspended (7.5 × 10^6^ cells/mL) in CLM-H with a range of free [Ca^2+^] and supplemented with CPA (10 μM). The cells were distributed into 96-well plates and after 30 s, ionomycin (10 μM) was added to accelerate the equilibration of Ca^2+^ between the ER lumen and medium. QAb (1:20) was included in all analyses of Mag-Fluo-4. We confirmed that QAb had no effect on the responses recorded from G-CEPIA1*er* (not shown). Fluorescence was recorded using a FlexStation III (Section 2.3).

### 2.6 Carboxylesterase assays and measurements of Mag-Fluo-4 affinity in vitro

Pilot reactions (150 μL) established optimal conditions for de-esterification of Mag-Fluo-4 AM by porcine liver carboxylesterase: 1 μM Mag-Fluo-4 AM and 1–1000 U/mL carboxylesterase in phosphate buffer (37.7 mM Na_2_HPO_4_, 12.3 mM NaH_2_PO_4_, pH 7.5) for 15 min at 37 °C. Variations in the durations of the incubations or concentrations of enzyme or Mag-Fluo-4 AM are reported in figure legends. At appropriate times, samples (5 μL) were removed and diluted into CLM-H containing various free [Ca^2+^] (50 μL) at 22 °C and fluorescence was determined immediately using a FlexStation III (Section 2.3). Control experiments used Mag-Fluo-4 tetrapotassium salt or carboxylesterase without indicator.

### 2.7 Analysis

For Ca^2+^ release assays and to estimate the apparent affinity of Ca^2+^ indicators, concentration-response curves were fitted to Hill equations from which pK_D_ or pEC_50_ (-log of K_D_ and half-maximally effective concentration, respectively) were determined. All statistical analyses used log values (pEC_50_ and pK_D_), although for greater clarity we also report EC_50_ and K_D_ values. Student’s *t*-test or one-way repeated ANOVA with Bonferroni's multiple comparison test was used with *P* < 0.05 considered significant (PRISM, version 5, GraphPad Software, La Jolla, CA, USA).

## 3 Results and discussion

### 3.1 Mag-fluo-4 reliably reports Ca^2+^ uptake and release from the ER

In permeabilized DT40-IP_3_R1 cells with Mag-Fluo-4 trapped within the ER lumen (Fig. 1A), MgATP stimulated Ca^2+^ uptake, reported by an increase in fluorescence intensity (Fig. 1B). The response was abolished by CPA (10 μM) added 60 s before MgATP (Fig. 1B). This indicates that the increase in fluorescence was entirely due to SERCA-mediated Ca^2+^ uptake. Addition of CPA to permeabilized cells loaded to steady state with Ca^2+^ unmasked a slow Ca^2+^ leak (Fig. 1C). Semi-logarithmic plots of the fluorescence intensity after addition of CPA (Fig. 1D) revealed that the half-time (t_1/2_) was 356 ± 52 s and the intercept at the time of CPA addition was 102.2 ± 0.9 % (mean ± SEM, *n* = 3). These results, which concur with our published data [13,28], suggest a linear relationship between fluorescence and ER free [Ca^2+^] across the entire range of ER Ca^2+^ contents. This is unexpected for an indicator with a reported K_D_^Ca^ of 22 μM [18] that should be saturated at a steady-state ER free [Ca^2+^] of between 100 μM and 1 mM [[14], [15], [16]].

Addition of IP_3_ after CPA to permeabilized DT40-IP_3_R1 cells loaded to steady state with Ca^2+^ caused a concentration-dependent Ca^2+^ release evinced by the decrease in luminal Mag-Fluo-4 fluorescence (Fig. 1E and F). In the same assay, adenophostin A, a high-affinity agonist of IP_3_R [31], was ∼10-fold more potent than IP_3_ (Fig. 1F). These results, which agree with those obtained using similar [13,[19], [20], [21]] or different methods [32], confirm that Mag-Fluo-4 trapped within the ER can reliably report IP_3_-evoked Ca^2+^ release from the ER.

To further validate the use of Mag-Fluo-4 as an ER luminal Ca^2+^ indicator, we compared the responses reported by Mag-Fluo-4 and G-CEPIA1*er*, a low-affinity GECI targeted to the ER lumen (K_D_^Ca^ = 672 ± 23 μM) [25]. In permeabilized HEK cells stably expressing G-CEPIA1*er* or with the ER loaded with Mag-Fluo-4, IP_3_ caused a concentration-dependent release of Ca^2+^ from the ER. The responses reported by each indicator were indistinguishable, with no significant differences in the values for pEC_50_, maximal response or Hill coefficient (*h*) (Fig. 1G and Table 1). These results demonstrate that even though the two indicators differ by ∼30-fold in their K_D_^Ca^ determined *in vitro,* each reliably reports ER free [Ca^2+^].

**Table 1:** Properties of Mag-Fluo-4 and G-CEPIA1*er.* Effects of IP_3_ on Ca^2+^ release from the intracellular stores of permeabilized HEK cells loaded with Mag-Fluo-4 AM or stably expressing G-CEPIA1*er* (from Fig. 1G). Results show means ± SEM (pEC_50_, Ca^2+^ release (%) and *h*) and means (EC_50_) from 6-9 independent experiments, each performed in duplicate. EC_50_, half-maximally effective concentration; pEC_50_, -logEC_50_; *h*, Hill coefficient. There are no significant differences between pEC_50_, Ca^2+^ release (%) and *h* values for Mag-Fluo-4 and G-CEPIA1*er.* Summary results (*n* = 5 independent experiments, each with 3 replicates) show K_D_^Ca^ values determined for indicators within the ER (Fig. 3). ^a^The first values report those determined after addition of ionomycin, while values in brackets are those determined before adding ionomycin (see Fig. 3). Results means ± SEM (pK_D_ and *h*) and means (K_D_). *h* values are significantly different (*P* < 0.05) for the two indicators. For each indicator, the values (pK_D_ and *h*) determined before or after addition of ionomycin were not statistically different.

	IP_3_-evoked Ca^2+^ release			^a^K_D_^Ca^	
	pEC_50_ **EC_50_ (nM)**	Maximal release (%)	*h*	pK_D_ **K_D_ (μM)**	*h*
Mag-Fluo-4	6.62 ± 0.08 **240**	72 ± 2	1.2 ± 0.3	2.94 ± 0.21 **1148** [2.88 ± 0.20] **1318**	0.42 ± 0.04 [0.53 ± 0.05]
G-CEPIA1*er*	6.72 ± 0.09 **191**	77 ± 2	1.2 ± 0.1	2.81 ± 0.02 **1549** [2.84 ± 0.02] **1445**	1.54 ± 0.36 [2.03 ± 0.44]

### 3.2 Antibody-quenching of fluorescence from Mag-Fluo-4 leaked from the ER

**Fig. 2:**
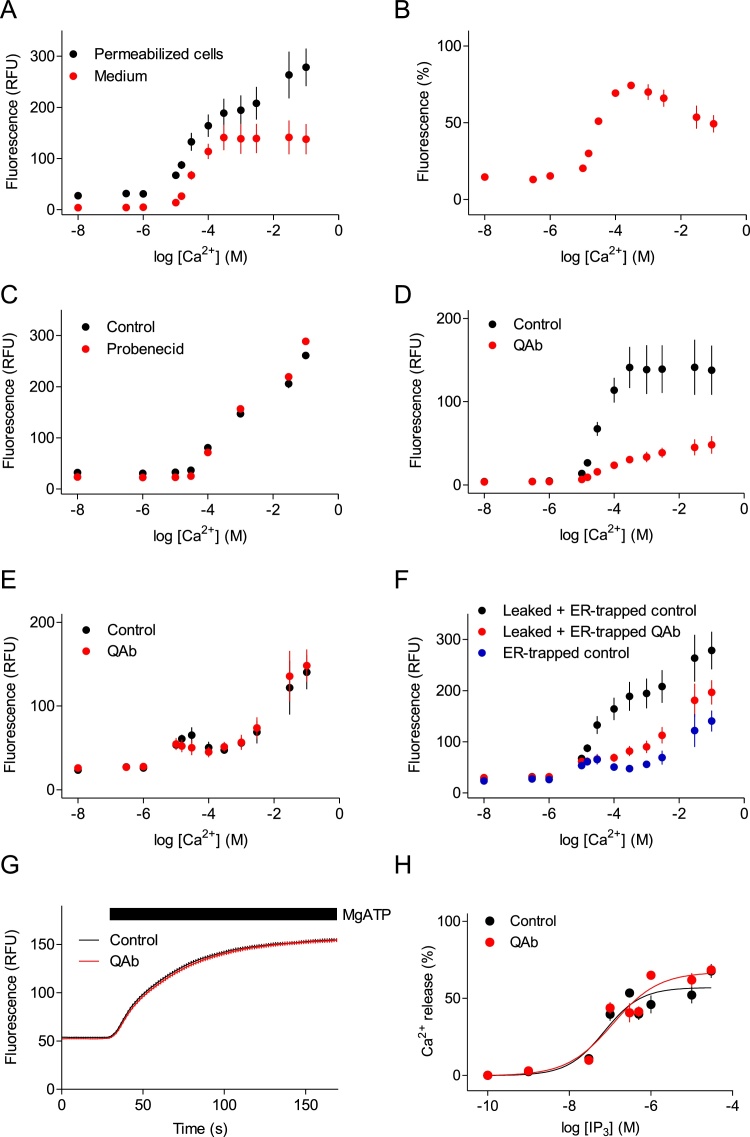
Fluorescence from leaked Mag-Fluo-4 is quenched by QAb. (A) Permeabilized HEK cells loaded with Mag-Fluo-4 AM were re-suspended in CLM-H (i.e. CLM supplemented with HEDTA to provide effective buffering of high free [Ca^2+^]), with the indicated [Ca^2+^]_m_ and CPA (10 μM). We note that with the prolonged incubations used (5-20 min), luminal [Ca^2+^] and [Ca^2+^]_m_ equilibrate without the need for ionomcyin to accelerate equilibration (see Fig. 3A). Results (means ± SEM, *n* = 3 independent experiments each with duplicate determinations) show parallel analyses of the medium alone (after removal of cells by centrifugation, F_medium_) or cells in the medium (F_permeabilized cells_). (B) Results from panel A were used to estimate the contribution of leaked indicator to total fluorescence intensity (F_medium_/F_permeabilized cells,_ %) at each [Ca^2+^]_m_. (C) Fluorescence recorded from permeabilized cells after treatment with probenecid (2.5 mM). Means ± SD from a single experiment, typical of two experiments, each with duplicate determinations. (D) Effects of QAb (1:20) on Ca^2+^-dependent changes in fluorescence from leaked Mag-Fluo-4 (F_medium_). Means ± SEM, *n* = 3 independent experiments, each with duplicate determinations (control trace reproduced from panel A). (E) Comparison of the responses of trapped Mag-Fluo-4 (F_permeabilized cells_ - F_medium_) to changes in [Ca^2+^]_m_ in the absence or presence of QAb (1:20). Means ± SEM, *n* = 3 independent experiments, each with duplicate determinations. (F) Effects of QAb (1:20) on Ca^2+^-dependent changes in fluorescence from permeabilized cells together with their medium (F_permeabilized cells_), or from trapped indicator alone (F_permeabilized cells_ - F_medium_) in the absence of QAb. Means ± SEM, *n* = 3 independent experiments each with duplicate determinations (blue symbols reproduced from panel E). The results confirm that most fluorescence from leaked indicator is quenched by QAb. (G) Ca^2+^ uptake by permeabilized cells loaded with Mag-Fluo-4 AM and incubated in CLM with free [Ca^2+^]_m_ = 300 nM was stimulated by addition of ATP (1.5 mM). QAb (1:20) had no effect on the response. (H) Concentration-dependent effects of IP_3_ on Ca^2+^ release from permeabilized HEK cells in CLM with 300 nM [Ca^2+^]_m_. QAb (1:20) had no effect. To minimize use of costly QAb, results in G and H (means ± SD) are from a single experiment with 3 replicates.

Parallel measurements of the K_D_^Ca^ for Mag-Fluo-4 and G-CEPIA1*er* within the ER were compromised because Mag-Fluo-4, like other Ca^2+^ indicators, leaks from the ER [11] and it is then exposed to the high [Ca^2+^]_m_ used for calibration. Fig. 2A illustrates the problem: increasing the free [Ca^2+^]_m_ in supernatants derived from cells in which the ER had been loaded with Mag-Fluo-4 caused concentration-dependent increases in fluorescence. The K_D_^Ca^ of this leaked Mag-Fluo-4 was ∼38 μM (pEC_50_ = 4.42 ± 0.07, *h* = 1.8 ± 0.3, *n* = 4). These values are similar to those we determined for Mag-Fluo-4 pentapotassium salt *in vitro* (K_D_^Ca^ = 16 μM, pK_D_^Ca^ = 4.79 ± 0.04, *h* = 1.21 ± 0.17, *n* = 3, see Fig. 4C and D) and with the published K_D_^Ca^ of ∼22 μM [18], suggesting that the leaked indicator is probably fully de-esterified [11].

To determine the contribution of leaked indicator to the measured fluorescence, permeabilized cells were treated with CPA to prevent active Ca^2+^ uptake and re-suspended in CLM-H with various [Ca^2+^]_m_. Cells were then split, with 50 % plated for fluorescence measurements and 50 % centrifuged to isolate the medium. Fluorescence intensities were measured for medium alone (F_medium_) and for cells in the medium (F_permeabilized cells_). Comparison of the fluorescence changes recorded from medium alone or from cells in medium shows that leaked indicator (F_medium_) responds to free [Ca^2+^] within the 1−300 μM range, while trapped indicator (the difference between F_permeabilized cells_ and F_medium_) responds most above 300 μM (Fig. 2A). From these results, we calculated the contribution of leaked indicator to overall fluorescence intensity. The contribution from the leaked indicator is maximal (∼75 % of overall fluorescence) when [Ca^2+^]_m_ is ∼300 μM. At higher [Ca^2+^]_m_ - as the contribution of trapped indicator, with its lower affinity for Ca^2+^, becomes larger (Fig. 2B) - the contribution from the leaked indicator decreases. These results illustrate the scale of the problem with leaked indicator, and they demonstrate that the indicator within the ER must bind Ca^2+^ with lower affinity than that in the medium.

We were unable to reduce the contribution from leaked indicator by washing cells (not shown), suggesting rapid re-equilibration after removal of Mag-Fluo-4 from the medium. Probenecid (2.5 mM), which blocks organic anion transporters and is often used to prevent loss of indicator from cells [22,33], was also ineffective (Fig. 2C). The fluorescence intensity recorded at saturating [Ca^2+^]_m_ from medium recovered from cells with probenecid treatment was 105 ± 7 % (*n* = 2) of that recovered from control cells. We therefore attempted to quench the fluorescence of leaked Mag-Fluo-4 using an anti-fluorescein/Oregon Green antibody (QAb) that has been shown to quench the fluorescence of Fluo-3 [34,35]. Permeabilized cells treated with CPA to prevent active Ca^2+^ uptake and medium separated from these cells were pre-incubated with QAb before measuring fluorescence. QAb significantly reduced fluorescence from the leaked indicator (by 70–80 % for [Ca^2+^]_m_ > 10 μM) (Fig. 2D), without affecting the fluorescence intensity of trapped Mag-Fluo-4 at any [Ca^2+^]_m_ (Fig. 2E). QAb reduced the fluorescence intensity to values that came close to those attributable to trapped indicator alone (determined from F_permeabilized cells_ – F_medium_) (Fig. 2E and F). We confirmed that QAb did not interfere with Ca^2+^ uptake (Fig. 2G) or IP_3_-evoked Ca^2+^ release (Fig. 2H). The former is important because it also demonstrates that QAb does not exaggerate the loss of indicator from the ER (Fig. 2G).

We conclude that quenching of leaked Mag-Fluo-4 by QAb allows the trapped indicator to be used with permeabilized cells in medium with high [Ca^2+^]_m_. QAb also provides the tool needed to allow the K_D_^Ca^ of indicator trapped within the ER to be determined.

### 3.3 Mag-Fluo-4 within the ER has low-affinity for Ca^2+^

**Fig. 3:**
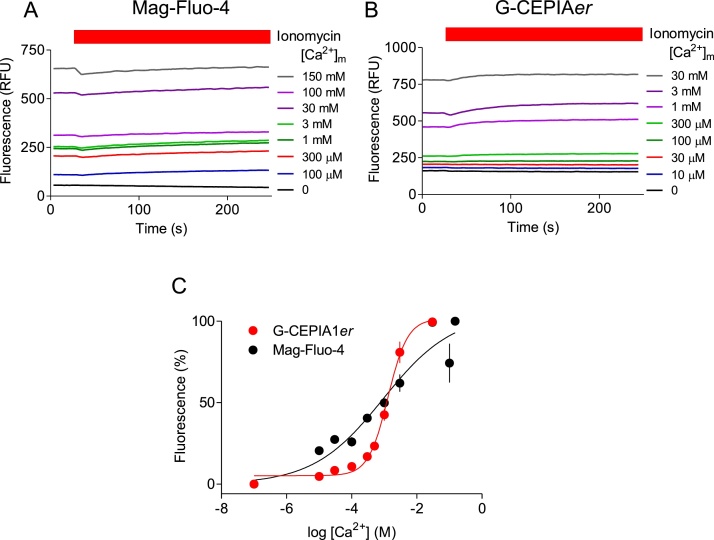
Mag-Fluo-4 within the ER has low-affinity for Ca^2+^. (A, B) Typical traces show the fluorescence recorded from permeabilized HEK cells pre-treated (30 min) with CPA (10 μM) in Ca^2+^-free CLM before re-suspending them in CLM-H with the indicated [Ca^2+^]_m_. The recordings begin 5-20 min after distributing the cells into 96-well plates. Ionomycin (10 μM) was added where indicated. K_D_^Ca^ values were determined from fluorescence values measured either before addition of ionomycin or during the last 20 s of the recording (Table 1). Typical traces are shown for cells loaded with Mag-Fluo-4 (A) or expressing G-CEPIA1*er* (B). (C) Ca^2+^-dependent changes in the fluorescence of Mag-Fluo-4 or G-CEPIA1*er* trapped within the ER of permeabilized HEK cells, determined after addition of ionomcyin. Means ± SEM, *n* = 5 independent experiments, each with 3 determinations. Summary results in Table 1.

To determine the K_D_^Ca^ of Mag-Fluo-4 and G-CEPIA1*er* within the ER, permeabilized cells loaded with Mag-Fluo-4 or stably expressing G-CEPIA1*er* were incubated with CPA to empty the intracellular stores and re-suspended in CLM-H with different [Ca^2+^]_m_. Ionomycin (10 μM) was then added to accelerate equilibration of luminal [Ca^2+^] with [Ca^2+^]_m_, and, for Mag-Fluo-4 (Fig. 3A and B), QAb (1:20) was included to quench the fluorescence of leaked indicator. The results establish that during the prolonged incubation (∼5−20 min) before adding ionomycin, the luminal [Ca^2+^] had already equilibrated with [Ca^2+^]_m_, since addition of ionomycin evoked no further changes in luminal fluorescence (Fig. 3A and B). The observation is important because it confirms that ionomycin does not perturb the calibration (by, for example, affecting luminal pH through H^+^/Ca^2+^ exchange). The results indicate K_D_^Ca^ values of 1.15 mM for Mag-Fluo-4 and 1.55 mM for G-CEPIA1*er*, and *h* of 0.42 and 1.54, respectively (Fig. 3C, Table 1, wherein the similar K_D_^Ca^ values determined without using ionomycin are also reported). The values for G-CEPIA1*er* are comparable to published values (K_D_^Ca^ = 672 μM, *h* = 1.95) [25], but the affinity of Mag-Fluo-4 determined within the ER is some 30 to 50-fold lower than the values determined *in vitro* by us (K_D_^Ca^ ∼38 μM; Fig. 2A) or others (K_D_^Ca^ = 22 μM) [18].

**Fig. 4:**
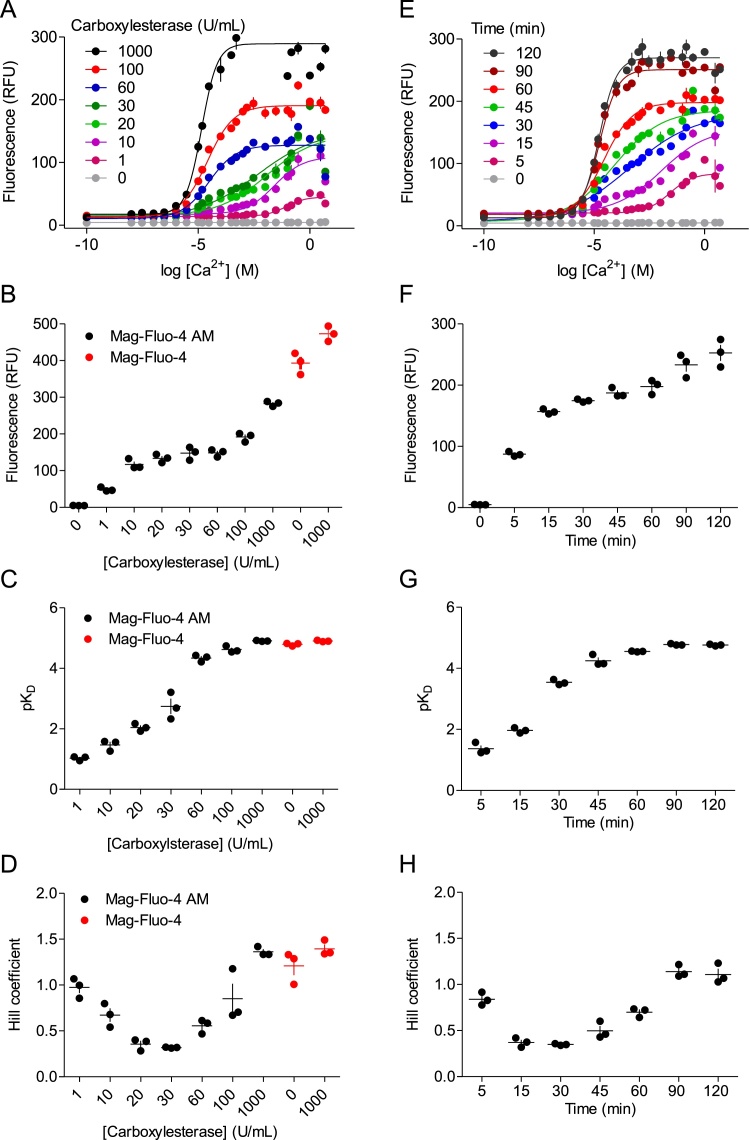
Partial de-esterification of Mag-Fluo-4 AM provides heterogenous indicator with low Ca^2+^ affinity. (A) Ca^2+^-dependent change in the fluorescence of Mag-Fluo-4 AM (0.1 μM) or Mag-Fluo-4 (0.1 μM) after incubation (15 min at 37 °C) with the indicated concentrations of carboxylesterase. (B) Effects of carboxylesterase concentration on the fluorescence intensity measured at saturating [Ca^2+^]_m_ (5 M). (C, D) Effects of carboxylesterase concentration on pK_D_^Ca^ (C) and *h* (D). (E) Ca^2+^-dependent change in the fluorescence of Mag-Fluo-4 AM (0.1 μM) after incubation (37 °C) with carboxylesterase (20 U/mL) for the indicated times. (F) Effects of incubation duration on the fluorescence intensity measured at saturating [Ca^2+^]_m_ (5 M). (G, H) Effects of incubation duration on pK_D_^Ca^ (G) and *h* (H). All results show means ± SEM, *n* = 3 independent experiments each with duplicate determinations; individual values are also shown in B-D and F-H.

These results indicate that Mag-Fluo-4 loaded into the ER by incubation of cells with Mag-Fluo-4 AM has lower affinity for Ca^2+^ than expected. Furthermore, the shallow Hill slopes (*h* = 0.42) suggest a heterogenous population of luminal indicator molecules with different affinities for Ca^2+^.

### 3.4 Partial de-esterification of Mag-Fluo-4 probably underlies its low affinity within the ER

The K_D_^Ca^ values of several Ca^2+^ indicators measured within the ER are 1.3–8.5-fold lower than values determined *in vitro* [11,18]. The differences have been attributed to differences in pH, temperature, ionic strength, viscosity, and the presence of proteins and other ions within the ER [17]. For Mag-Fluo-4, the K_D_^Ca^ determined within the ER is about 50-fold greater than that determined *in vitro* (Fig. 3), suggesting that other factors may contribute. Furthermore, the relationship between free [Ca^2+^] and fluorescence is much shallower for Mag-Fluo-4 within the ER (*h* < 1) (Fig. 3), suggesting the presence of Ca^2+^-binding sites with heterogenous affinities. These observations prompted us to consider whether the luminal indicator might be only partially de-esterified.

Mag-Fluo-4 AM was incubated *in vitro* with carboxylesterase, and fluorescence was measured at different [Ca^2+^]_m_. As expected, Mag-Fluo-4 AM was not fluorescent at any [Ca^2+^]_m_ (Fig. 4A and E), consistent with it being unable to bind Ca^2+^. When Mag-Fluo-4 AM was incubated with carboxylesterase, the fluorescence recorded at saturating [Ca^2+^]_m_ increased (Fig. 4A and B), confirming that the enzyme de-esterified Mag-Fluo-4 AM to forms that bind Ca^2+^. The affinity of the indicator for Ca^2+^ increased with increasing amounts of carboxylesterase (Fig. 4C). At the highest enzyme concentration (1000 U/mL), the K_D_^Ca^ (12.3 μM) was similar to the K_D_^Ca^ of Mag-Fluo-4 pentapotassium salt (16.2 μM) (Fig. 4C) and the published K_D_^Ca^ for Mag-Fluo-4 (22 μM) [18]. The slope of the relationship between [Ca^2+^]_m_ and fluorescence (reported by *h*) changed with enzyme concentration (Fig. 4D): *h* was 0.97 ± 0.06 at the lowest enzyme concentration (1 U/mL), it decreased with increasing enzyme concentrations (10–100 U/mL) to a minimal value of 0.32 ± 0.01 (with 30 U/mL), and *h* then increased towards a maximal value of 1.40 ± 0.03 with further increases in enzyme concentration. The *h* value obtained with the greatest enzyme concentration (1000 U/mL) was similar to that observed for Mag-Fluo-4 pentapotassium salt (*h* = 1.20 ± 0.01) (Fig. 4D). Incubation of Mag-Fluo-4 pentapotassium salt with carboxylesterase (1000 U/mL) had no effect on the maximal fluorescence, K_D_^Ca^ or *h* (Fig. 4B–D), confirming that the effects of the enzyme were selective for Mag-Fluo-4 AM. Similar results, namely a decrease in K_D_^Ca^ and an inverted bell-shaped change in *h*, were obtained by increasing the duration of the incubation of Mag-Fluo-4 AM with a fixed concentration of carboxylesterase (Fig. 4E-H).

Our results confirm both the K_D_^Ca^ of Mag-Fluo-4 measured *in vitro* (∼20 μM) (Fig. 4C) and that sufficient treatment of Mag-Fluo-4 AM with carboxylesterase fully converts it to a Ca^2+^-sensitive form indistinguishable from Mag-Fluo-4 (Fig. 4). However, partial de-esterification of Mag-Fluo-4 AM produced a heterogenous population of Mag-Fluo-4 species that bind Ca^2+^ with reduced affinity. Incubation of Mag-Fluo-4 AM with the lowest concentration of carboxylesterase used (1 U/mL) produced a homogenous population of indicator (*h* = 0.97 ± 0.06) that bound Ca^2+^ with extremely low affinity (K_D_^Ca^ ∼ 93 mM). This species is not unmodified Mag-Fluo-AM since fluorescence of the latter is entirely insensitive to Ca^2+^ (Fig. 4A, B, E and F). Three carboxylate groups participate in coordinating Ca^2+^ in Mag-Fluo-4, and all three are esterified in Mag-Fluo-4 AM (Fig. 1A). We suggest that the very low-affinity species is probably the first product of de-esterification capable of binding Ca^2+^. Higher concentrations of enzyme (10–100 U/mL) or more prolonged incubations provides a heterogenous population of Ca^2+^-binding sites (*h* < 1) with average affinities that increase with enzyme concentration (K_D_^Ca^ =34 mM - 24 μM) or incubation time. We have not resolved whether mixtures of only the two forms of indicator, namely the very-low affinity (K_D_^Ca^ =93 mM), partially de-esterified form and Mag-Fluo-4 itself (K_D_^Ca^ ∼20 μM), are sufficient to explain our results.

**Fig. 5:**
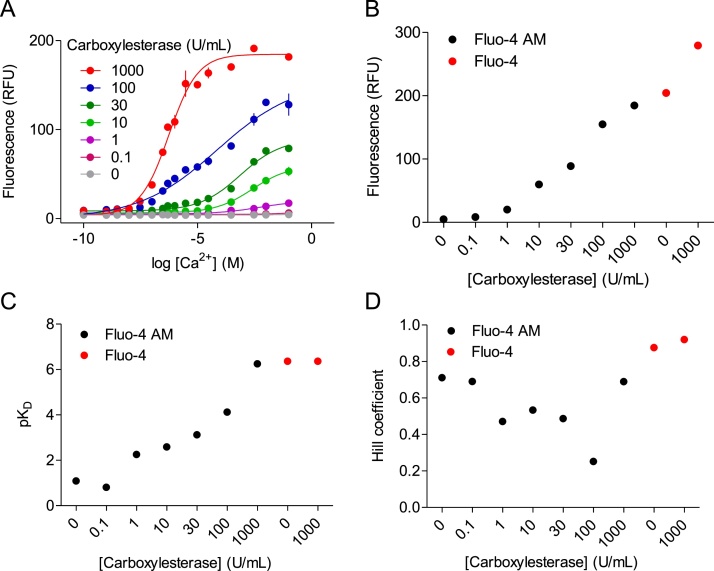
Partial de-esterification of Fluo-4 AM provides heterogenous indicator with low Ca^2+^ affinity. (A) Ca^2+^-dependent change in the fluorescence of Fluo-4 AM (0.1 μM) or Fluo-4 (0.1 μM) after incubation (15 min at 37 °C) with the indicated concentrations of carboxylesterase. Results show mean ± SD from 1 experiment with duplicate determinations. (B-D) Effects of carboxylesterase concentration on the fluorescence intensity measured at saturating [Ca^2+^]_m_ (100 mM), pK_D_^Ca^ (C) and *h* (D). Results are means from 1 experiment with duplicate determinations.

Analyses of other esterified Ca^2+^ indicators, namely 5F-BAPTA [36] and Fura-2 [37,38], concluded that partially de-esterified forms do not bind Ca^2+^, but the highest [Ca^2+^]_m_ used in these studies were too low (∼300 nM [36], 100 μM [37] and ∼1 mM [38]) to identify very low-affinity Ca^2+^-binding sites. The Ca^2+^-binding site of indicators like Fura-2, 5F-BAPTA and Fluo-4 is based on BAPTA (1,2-bis(O-aminophenoxy)ethane-N,N,N′,N′-tetraacetic acid), wherein four carboxylate groups coordinate Ca^2+^ [39]. Mag-Fluo-4, however, is based on APTRA (aminophenol triacetic acid) with three carboxylate groups [40] (Fig. 1A). BAPTA and APTRA (and the indicators derived from them) coordinate Ca^2+^ differently: one BAPTA molecule coordinates a single Ca^2+^ ion [39], whereas a pair of APTRA molecules together coordinate two Ca^2+^ ions [40]. We therefore considered whether Ca^2+^ indicators built around BAPTA and APTRA might differ in whether their partially de-esterified forms respond to Ca^2+^. However, preliminary studies suggest that the effects of incubating Fluo-4 AM (a BAPTA-based indicator) with carboxylesterase to cause partial de-esterification are similar to those we observed with Mag-Fluo-4 AM, namely a progressive decrease in K_D_^Ca^ towards that of the fully de-esterified indicator, and a heterogenous population of Ca^2+^-binding sites (*h* < 1) after partial de-esterification (Fig. 5). We suggest that many Ca^2+^ indicators, whether based on APTRA or BAPTA, may bind Ca^2+^ with low and heterogenous affinities in their partially de-esterified forms.

## 4 Conclusions

We have confirmed that Mag-Fluo-4, loaded into cells in its AM form, reliably reports changes in free [Ca^2+^] within the ER of permeabilized cells (Fig. 1), despite its high affinity measured *in vitro* (K_D_^Ca^ ∼20 μM) (Fig. 4C). Using an antibody (QAb) to selectively quench indicator that leaks from the ER, we demonstrated that the Ca^2+^ affinity of indicator trapped in the ER is much lower (K_D_^Ca^ ∼1.15 mM) and more heterogenous (*h* = 0.42) than Mag-Fluo-4 (Fig. 3C). Our analyses of the effects of carboxylesterase on Mag-Fluo-4 AM showed that partially de-esterified forms of the indicator mimic the properties of indicator within the ER (Fig. 4): both have low and heterogenous affinity for Ca^2+^.

Three features of Mag-Fluo-4 AM behaviour fortuitously contribute to its utility as a reliable reporter of ER free [Ca^2+^]. Firstly, partial de-esterification of Mag-Fluo-4 AM provides an average K_D_^Ca^ appropriate for an organelle with a steady-state free [Ca^2+^] between 100 μM and 1 mM [[14], [15], [16]]. Secondly, a mixture of partially de-esterified forms of indicator with different K_D_^Ca^ (reflected in the low values for *h*) provides sensitivity across a wide range of free [Ca^2+^] (Fig. 3). Finally, the ER selectively exports the fully de-esterified form of the indicator (Fig. 2A) by mechanisms that are not inhibited by probenecid (Fig. 2C). This leakage creates problems for experiments that require measurements in media with substantially increased [Ca^2+^]_m_, but our use of a quenching antibody (QAb) substantially ameliorates the problems without affecting biological responses (Fig. 2G and H) or retention of ER-localized indicator (Fig. 2G). More importantly, selective extrusion of indicator with inappropriately high affinity ensures that fluorescence from the partially de-esterified forms, with their useful low affinity, dominates the signal from the ER lumen.

We conclude that partial de-esterification of Mag-Fluo-4 AM within the ER alongside extrusion of the fully de-esterified form allow the indicator to reliably report changes in ER free [Ca^2+^].

## Author contributions

A.M.R. performed all experiments. A.M.R and C.W.T. designed the study, analysed and interpreted results, and wrote the manuscript.

## Declaration of Competing Interest

The authors declare they have no competing interests.
